# Using multiple imputation and intervention-based scenarios to project the mobility of older adults

**DOI:** 10.1186/s12877-022-03008-4

**Published:** 2022-04-09

**Authors:** Jukka Kontto, Laura Paalanen, Reijo Sund, Päivi Sainio, Seppo Koskinen, Panayotes Demakakos, Hanna Tolonen, Tommi Härkänen

**Affiliations:** 1 Department of Public Health and Welfare, Finnish Institute for Health and Welfare (THL), P.O. Box 30, FI-00271 Helsinki, Finland; 2 Institute of Clinical Medicine, University of Eastern Finland, Kuopio, Finland; 3 Department of Epidemiology and Public Health, University College London, London, UK

**Keywords:** Mobility limitations, Older adults, Randomised controlled trial, Projection, Multiple imputation

## Abstract

**Background:**

Projections of the development of mobility limitations of older adults are needed for evidence-based policy making. The aim of this study was to generate projections of mobility limitations among older people in the United States, England, and Finland.

**Methods:**

We applied multiple imputation modelling with bootstrapping to generate projections of stair climbing and walking limitations until 2026. A physical activity intervention producing a beneficial effect on self-reported activities of daily living measures was identified in a comprehensive literature search and incorporated in the scenarios used in the projections. We utilised the harmonised longitudinal survey data from the Ageing Trajectories of Health – Longitudinal Opportunities and Synergies (ATHLOS) project (*N* = 24,982).

**Results:**

Based on the scenarios from 2012 to 2026, the prevalence of walking limitations will decrease from 9.4 to 6.4%. A physical activity intervention would decrease the prevalence of stair climbing limitations compared with no intervention from 28.9 to 18.9% between 2012 and 2026.

**Conclusions:**

A physical activity intervention implemented on older population seems to have a positive effect on maintaining mobility in the future. Our method provides an interesting option for generating projections by incorporating intervention-based scenarios.

**Supplementary Information:**

The online version contains supplementary material available at 10.1186/s12877-022-03008-4.

## Background

The future development of functional limitations among older people is of particular interest since the population is rapidly ageing globally [1]. According to several studies, the projected prevalence of functional limitations, e.g. restrictions in activities of daily living (ADL) or instrumental activities of daily living (IADL), is expected to increase in the future [2–4]. However, the prevalence of functional limitations of older people varies between and within countries over time [5, 6]. The consequences of increase in functional limitations are increased health care costs, lower quality of life, and premature mortality [7–12]. The information on future development of functional limitations is essential for societies’ ability to meet the challenges of population ageing [13].

In previous research, the increase in healthcare and community care resources and rehabilitation have been proposed as possible attempts to mitigate the burden of functional limitations [14–16]. Also, interventions targeting chronic conditions associated with functional limitations [17, 18] and healthy behaviour, such as physical activity and nutrition [16], have been suggested. An integral part of evidence-based policy making when, for instance, planning and implementing preventive actions is to generate projections of the future prevalence of functional limitations. The most efficient actions can be determined by incorporating the actions as scenarios to the projections and generating future estimates under each scenario.

Scenarios can serve different purposes and be based on pre-defined goals [19] or relevant health policy actions [20, 21]. Scenarios may also be based on a theoretical change in the determinants (such as obesity) with the extreme case of this being scenarios impossible or at least difficult to achieve, for example “all individuals become non-smokers and normal-weight”, in which case they serve best in identifying the range of possible projection estimates [22–24]. Another possibility to define scenarios is to utilize the results observed of real-life interventions aiming to improve health or functioning, and to estimate this effect in the population. Randomised controlled trials (RCTs) provide high-quality evidence of the effectiveness of interventions [25]. Using results from RCTs, scenarios can be created by incorporating this evidence as an estimate of the effectiveness which could be expected if the intervention was carried out at the population level.

In the current study, we projected the future prevalence of mobility limitations. Mobility is an integral part of functioning since mobility is essential when carrying out daily self-care activities and taking part in civic life [26]. Mobility limitations are also associated with health problems and injuries, social isolation, disability and dependence [26–28]. We used a novel non-parametric projection method accounting for possible interactions and non-linear relationships between variables. The method exploited longitudinal survey data to project not only individual-level changes in the outcome but also in the risk factors. The aims of this study were to: 1) generate projections for the prevalence of mobility limitations of older people until 2026 utilizing longitudinal survey data from three countries and 2) incorporate alternative scenarios exploiting interventions found from research literature.

## Methods

### Literature review on RCT results

We performed a literature search to identify suitable RCTs aimed at improving the functioning of older people, and incorporated information from them as alternative scenarios to the projections. A literature search was conducted by an information specialist at the Finnish Institute for Health and Welfare in April 2019. As the sample sizes in individual RCTs tend to be small, the literature search focused on reviews and meta-analyses. The search strategy is presented in Additional file 1.

Finally, the meta-analysis by Roberts et al. [29] with ADL and IADL as the outcome was selected. The objective of the meta-analysis was to evaluate the effect of physical activity interventions on ADL and IADL measures. The meta-analysis included 47 RCTs with participants with a minimum age of 55 years and a mean age ≥ 60 years. The type of physical activity varied between the studies. Multicomponent interventions, e.g. interventions including a nutritional component in addition to physical activity were not included in the meta-analysis.

### Scenarios

We utilized the following result from the meta-analysis by Roberts et al. [29] for creating scenarios: the intervention group with moderate physical activity level (vs. no activity control group) produced a beneficial effect on self-reported ADL and IADL measures with standardised mean difference (SMD) 1.12, 95% confidence interval (CI) (0.74, 1.49) and *p*-value < 0.01. This was accomplished by pooling four studies, in which the intervention durations varied between 2 and 8 months.

For the current study, we defined the following three scenarios:

- Scenario 0:
  No physical activity intervention

- Scenario 1:
  Physical activity intervention produced beneficial effect on IADL score with SMD 1.12

- Scenario 2:
  Physical activity intervention produced beneficial effect on both IADL and ADL scores with SMD 1.12.

We projected the prevalence of mobility limitation outcomes under these three scenarios. The effect of the physical activity intervention on IADL and ADL score was incorporated in scenarios 1 and 2. If there was an association between IADL and ADL score and mobile limitation outcomes, the physical activity intervention was assumed to have effect on mobility limitations. In previous research, physical activity has been found to affect mobility limitations of older adults [30, 31].

### The ATHLOS dataset

The analysis dataset was constructed by exploiting the harmonised data of three studies from the Ageing Trajectories of Health – Longitudinal Opportunities and Synergies (ATHLOS) project [32]:

#### The Health and Retirement Study (HRS)

The initial HRS cohort was recruited in 1992 and consisted of residents of the United States of America aged 51–61 years [33]. At baseline, the number of participants was 12,652 (participation rate 81.6%), and the cohort has been followed up biennially. The follow-up participation rates among baseline participants who were alive and had not requested to be removed from the sample varied between 85.4 and 89.6% from 1994 to 2014 [34]. Data collections 2000–2012 were included in the analysis dataset.

#### The English Longitudinal Study of Ageing (ELSA)

ELSA was developed as a companion study to HRS and designed to represent the older population of England. The baseline cohort consisted of community-dwelling individuals aged ≥50 years in 2002 [35]. The number of participants at baseline was 12,099 including 11,391 sample members and 708 partners with the participation rate of sample members being 67%. The cohort has been followed up biennially with the participation rates conditional upon participating in 2002 decreasing from 82% in 2004 to 66% in 2012 [36]. The cohort was refreshed in 2006, 2008, 2012, 2014, and 2018 to maintain the representativeness of the sample [37]. Data collections 2002–2012 were included in the analysis dataset.

#### The Health 2000 and Health 2011 Surveys (H2000)

The Health 2000 Survey is a health examination survey conducted in Finland in 2000 with 9125 participants aged ≥18 [38]. The sample included 4084 participants aged ≥50 with their participation rate 92.8%. The follow-up survey was performed in 2011 including 3669 participants from the eligible Health 2000 sample who were aged ≥50 with the participation rate 77.4% [39].

### Variables extracted from the ATHLOS dataset

#### Self-reported ADL and IADL measures

Utilizing the harmonized ATHLOS variables we defined an ADL score [40], which consisted of six functions (ADL items): bathing or showering, getting dressed, using the toilet, getting in or out of bed, continence, and eating. The question on difficulties in getting dressed was not available in HRS. We also defined an IADL score [41] which consisted of six functions (IADL items): making telephone calls, shopping groceries, preparing meals, doing housework, taking medications, and managing money. The question on difficulties in managing money was not available in H2000. The original IADL score also includes doing laundry and mode of transportation, but these variables were not harmonised in ATHLOS. The responses were dichotomised as having difficulties (coded as 0) versus no difficulties (coded as 1). Scores with scales from zero to six were calculated as the sum of the points, with zero indicating severe disability. ADL and IADL items were included in the analysis dataset, with ADL and IADL scores derived after the imputation of the analysis dataset (see Statistical methods section).

#### Mobility limitation outcomes

We selected mobility limitation outcomes utilizing the results by Caballero et al. [42] who developed a health metric in the ATHLOS project. In their procedure, 45 items related to functioning, and available in the ELSA baseline data, were assessed with exploratory factor analysis. As a result, five factors were identified representing the following dimensions of functioning: mobility, eyesight, cognitive functioning, psychological functioning, and hearing. We selected two items with the highest factor loading from the mobility factor with two preconditions: the item was available for all three studies (HRS, ELSA, H2000) and the item was not among ADL or IADL items. The following items were selected as outcomes: 1) Limitations in climbing one flight of stairs; 2) Limitations in walking across a room. Both outcomes were based on self-reported information. The results of outcomes representing other dimensions of functioning are presented in Additional file 1.

#### Mortality status

Mortality follow-up in HRS was obtained through two sources: 1) contacts to all previously surviving panel members or their next-of-kin before the next data collection, and 2) the National Death Index (NDI) [43]. The death information from latest NDI linkage was available until the end of 2011, while the death information from contacting next-of-kins was available until the end 2013. Overall, contacting next-of-kins identified 95.9% of deaths, so the mortality information was essentially complete in HRS through 2013 [43]. Mortality follow-up in ELSA was obtained from the National Health Service (NHS) Central Register until February 2012 [44]. Mortality follow-up in H2000 was not harmonised in ATHLOS but was obtained until 2017 directly from Statistics Finland.

#### Factors associated with mobility limitations

Smoking, obesity, alcohol use, hypertension, and physical activity were harmonized in the ATHLOS dataset and have previously been found to be associated with mobility limitations among the older adults [30, 31, 45–53]. Smoking (yes, no) was based on the question on whether a participant was smoking at the time of the survey. Obesity (body mass index ≥30 kg/m^2^) was based on measured weight and height in ELSA and H2000. Self-reported information on height and weight was used in HRS, and in H2000, if the measurements were missing. Alcohol use (yes, no) was based on the question on alcohol use in the preceding 12 months in ELSA and H2000, while in HRS the question ‘Do you ever drink any alcoholic beverages such as beer, wine, or liquor?’ was used. The variable was dichotomized as yes (any alcohol use) versus no (teetotaller). In ELSA and H2000, arterial hypertension (yes, no) was based on measured systolic and diastolic blood pressure. An individual was categorized as hypertensive if the mean of systolic blood pressure measurements was at least 140 mmHg, or the mean of diastolic blood pressure measurements was at least 90 mmHg, or both. Information on the use of antihypertensive medication was not included in the harmonization. In HRS, arterial hypertension was based on the question ‘Has a doctor ever told you that you have high blood pressure or hypertension?’. Vigorous physical activity (yes, no) was based on self-reported information, with those reporting an activity of least once a week were considered to have been engaged to vigorous physical activity. In HRS surveys 2000 and 2002 the cut-off was three times a week or more due to the difference in questionnaires. The information on vigorous physical activity was not available in the Health 2011 Survey, so the question on engagement in physical activity in general was used. Obesity and arterial hypertension were not available in ELSA 2002, 2006, and 2010 surveys.

#### Background variables

Age was included as a continuous variable, and sex, education and study (HRS, ELSA, or H2000) as categorical variables. For ELSA all age values over 90 were coded as 99 to protect participants’ anonymity. Education was defined as the highest formal education achieved classified as low (primary education or less), medium (secondary education), and high (tertiary education).

### Analysis dataset

The analysis dataset consisted of biennial longitudinal population survey data from 2000 to 2012 of HRS, ELSA, and H2000. The values of outcomes, ADL and IADL items, mortality status, risk factors, and background variables across seven waves along with time-independent variable study were included in the analysis dataset. In order to have compatible years across studies, the participants of the Health 2011 Survey were randomly assigned into years 2010 and 2012. Values of 2000 for ELSA, and 2002–2008 for H2000 were set to missing values. The analysis dataset was restricted to individuals who were at least 50 years old in 2000, and who had participated in the 2000 survey (2002 survey for ELSA). The analysis dataset consisted of 24,982 individuals (13,750 women and 11,232 men). The baseline characteristics of the variables are presented in Table 1.

**Table 1:** The characteristics of variables of the first survey of each study extracted from the ATHLOS cohort to the analysis dataset (*N* = 24,982)

Variable	HRS^**a**^	ELSA^**a**^	H2000^**a**^
Baseline wave	2000	2002	2000
Number of individuals	9765	10,815	4402
Age (mean)	62.9	66.0	66.1
Sex (%, men)	45.4	45.7	42.2
Education (% with low education)	25.3	48.1	60.3
**Mobility limitation outcomes**			
Stair climbing (% with limitations)	14.9	15.8	22.9
Moving (% with limitations)	4.8	3.5	10.0
**ADL items**			
Bathing or showering (% with limitations)	4.6	12.9	11.0
Getting dressed (% with limitations)		13.8	12.8
Using the toilet (% with limitations)	4.3	3.8	6.6
Getting in or out of bed (% with limitations)	5.6	6.9	13.6
Continence (% with limitations)	15.4	15.7	21.3
Eating (% with limitations)	2.2	1.9	4.5
**IADL items**			
Making telephone calls (% with limitations)	2.5	1.9	7.0
Shopping groceries (% with limitations)	6.5	9.9	17.1
Preparing meals (% with limitations)	4.2	4.6	15.2
Doing housework (% with limitations)	31.4	16.8	39.8
Taking medications (% with limitations)	2.2	1.6	6.4
Managing money (% with limitations)	3.7	2.6	
**Factors associated with mobility limitations**			
Smoker (%)	18.3	17.3	20.4
Obesity (%)	28.1	28.7^b^	27.0
Alcohol use (%)	48.1	87.8	69.2
Arterial hypertension (%)	45.4	41.2^b^	58.2
Vigorous exercise (%)	46.7	26.7	51.4

^*a*^*HRS* The Health and Retirement Study, *ELSA* The English Longitudinal Study of Ageing, *H2000* The Health 2000 and 2011 Surveys

^b^Prevalence of ELSA survey 2004 are presented since obesity and arterial hypertension were not available for ELSA survey 2002

### Statistical methods

#### Overview

We projected the prevalence of stair climbing and walking limitations under three scenarios. The projections were generated sequentially using multiple imputation (MI) for every 2 years from 2014 to 2026 after utilizing MI to analysis dataset. The intervention effect (scenarios 1 and 2) was incorporated when generating values for 2014. Our method was presented by Härkänen et al. [23].

#### The bootstrap and MI

Our projections were based on 120 bootstrap samples generated to handle the sampling variability, including that of the covariates, with survey sampling based on individuals utilizing the bootstrap method by Rao and Wu [54]. For each bootstrap sample we imputed the missing values in the sample, incorporated the scenarios, and generated scenarios until 2026. Finally, we combined the results of bootstrap samples. The multiple imputation (MI) was based on the chained equations and classification and regression trees (CART) [55, 56] using the R software [57]. We used CART to reduce the number of variables included in the imputation model to decrease the computation time. After imputations, ADL score and IADL score were derived and included in the imputed dataset replacing the individual ADL and IADL items. Then, we used CART again to select predictors for survey variables in 2012. Age, sex, and education in 2012 were forced as predictors in all imputation models. The imputation process is described in more detail in Additional file 1.

#### Incorporating scenarios and generating projections

**Fig. 1:**
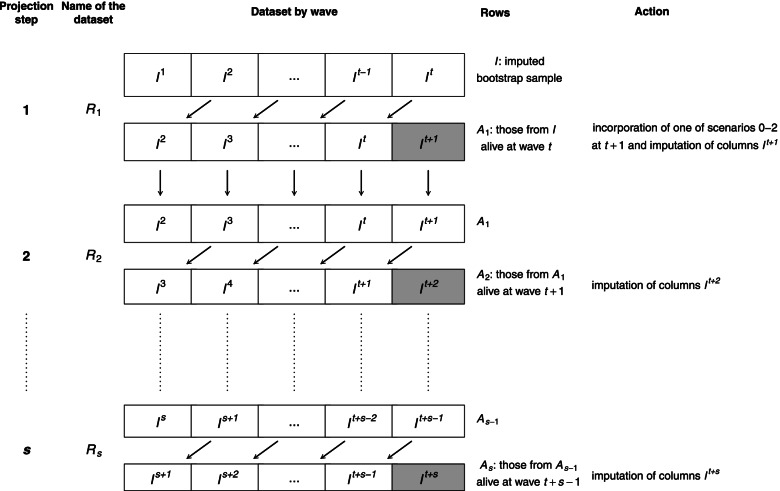
The dataflow of generating projections and datasets used in in each projection step. Projections are generated sequentially by imputing the values in the dataset marked with grey color in each step

The imputed individual-level dataset was denoted by *I* = (*I*^1^, …, *I*^*t*^) where waves 1, …, *t* = 7 corresponded the years 2000–2012 biennially, and the sequential projection step was indexed by *s*. The projection steps and dataflow are presented in Fig. 1.

At the first projection step (*s* = 1) the dataset *R*_1_ was the combination of the matrices ${\left({\mathrm{I}}^{\mathrm{T}},{\mathrm{A}}_{1}^{\mathrm{T}}\right)}^{\mathrm{T}}$ defining ${A}_1:= {\left({I}^2,\dots, {I}^{t+1}\right)}_{i:{d}_{i,t}=1,\cdot }$ the subset of *I*, where an individual *i* was alive at wave *t*, where values of *I*^*t* + 1^ were set to missing values, except age and sex, which remained constant or changed deterministically.

Then a copy of *R*_1_ was generated for each scenario and the scenarios are incorporated as follows:

- Scenario 0:

The missing values of *I*^*t* + 1^ in *A*_1_ were imputed with no modification of IADL or ADL scores.

- Scenario 1:

First, the missing values of *I*^*t* + 1^ were imputed (except outcomes and mortality status). Then, the SMD estimate 1.12 was transformed to the absolute scale of IADL score by multiplying the SMD estimate with the study-specific standard deviation (SD) of the IADL score in 2000 [*SD*(*y*_2000_)]. IADL score values for 2014 (*y*_2014_) of individuals with no vigorous physical activity were replaced with a random number from a normal distribution

$$N\left({y}_{2014}+1.12\times SD\left({y}_{2000}\right), SD\left({y}_{2000}\right)\right).$$

Finally, outcomes and mortality status in *I*^*t* + 1^ were imputed.

- Scenario 2:

Scenario 2 followed the same procedure as in scenario 1, but in addition to the modification of IADL scores, also the ADL score values for 2014 (*z*_2014_) of individuals with no vigorous physical activity were replaced, using the study-specific SD of the IADL score at 2000 [*SD*(*z*_2000_)], with a random number from a normal distribution

$$N\left({z}_{2014}+1.12\times SD\left({z}_{2000}\right), SD\left({z}_{2000}\right)\right).$$

In scenarios 1 and 2, the modified IADL and ADL scores below 0 were coded as 0, and scores over 6 were coded as 6.

In all scenarios *I*^*t* + 1^ was imputed using *R*_1_ with predictors in the imputation model. The assumption was that the survey variables will change between 2002 and 2014 with the same transition probabilities as between 2000 and 2012.

At the second projection step (*s* = 2) the dataset (*R*_2_) is the combination of the matrices ${\left({\mathrm{A}}_1^{\mathrm{T}},{\mathrm{A}}_2^{\mathrm{T}}\right)}^{\mathrm{T}}$ defining ${A}_2:= {\left({I}^3,\dots, {I}^{t+2}\right)}_{i:{d}_{i,t+1}=1,\cdot }$ the subset of *A*_1_, where an individual *i* was alive at wave *t* + 1 and the missing values of *I*^*t* + 2^ were imputed for all scenarios. Projection steps were repeated until we had imputed all the necessary values of *I*^*t* + *s*^ of the desired projection. The theory behind generating projections is described in Additional file 1.

#### Reporting the results

As our projections were generated using 120 bootstrap samples, both the expected value and the predictive variance of any statistic were calculated as the averages and variances, respectively, of the Monte Carlo simulations [58]. The predictors with selection proportion (the proportion of bootstrap samples a predictor was selected in the imputation model) of at least 10% were reported. We compared the projections between scenarios for each outcome pairwise by calculating the contrasts using of the differences of the point estimates. We report the 95% credible intervals (CrIs), and 95% CIs for the observed data. We present the results for individuals aged 76 years or older, since all individuals in the analysis dataset were at least 50 years old in 2000, so the minimum of age in 2026 was 76 years.

## Results

### Selection of predictors

The predictors with the highest selection proportion were the previous measurements of the corresponding outcome, with measurements 2002–2010 for stair climbing limitations, and measurements 2008–2010 for walking limitations (Table 2). IADL and ADL score in 2012 were predictors for both outcomes. For stair climbing limitations, the selection proportions of IADL score and ADL score in 2012 were 100 and 83%, respectively. For walking limitations, the result was opposite as the selection proportions were 100% for ADL score in 2012 and 57% for IADL score in 2012. Some of IADL and ADL scores 2008–2010 were also predictors for stair climbing limitations, with the selection proportions varying between 13 and 19%. The selection proportion of variable study was 45% for stair climbing limitations. When comparing the years of selected predictors, the predictors of stair climbing were concentrated on 2010–2012, while walking limitations had predictors from 2008 to 2012. Both stair climbing and walking limitations had predictors in 2012 with high selection proportion.

**Table 2:** The predictors of mobility limitation outcomes in 2012 by survey year. Numbers in parentheses are the proportions of bootstrap samples in which a variable was selected as a predictor. Variables with selection proportions at least 10% are included in the table. Age, sex, and education in 2012 are forced as predictors to all imputation models

Predictors^a^	Outcome in 2012	
	Stair climbing limitations	Moving limitations
**2012**	IADL (100) moving (86) ADL (83) VPA (16)	ADL (100) climbing (63) IADL (57)
**2010**	climbing (100) IADL (19) ADL (18) education (10)	moving (88)
**2008**	climbing (46) IADL (13)	moving (15)
**2006**	climbing (77)	
**2004**	climbing (20)	
**2002**	climbing (12)	
**2000**		
**time-independent**	study (45)	

^a^*IADL* IADL score, *ADL* ADL score, *VPA* vigorous physical activity; climbing = stair climbing limitations; moving = moving limitations

### Projections until 2026

#### Projected prevalence under scenario 0

**Fig. 2:**
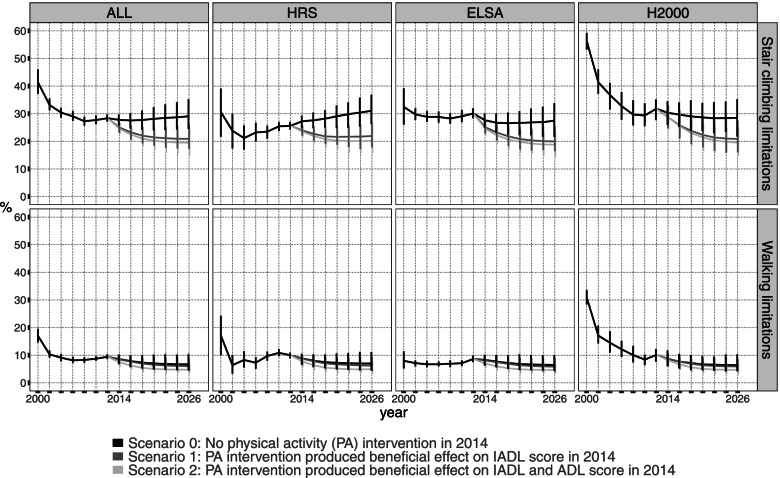
Projections of mobility limitation outcomes until 2026 by scenario and study

The prevalence of walking limitations will decrease between 2012 and 2026 (from 9.4% [CI 8.6, 10.3%] to 6.7% [CrI 4.6, 10.5%]) (Fig. 2, Table 3) due to the decrease in prevalence in HRS and H2000. For stair climbing limitations, the prevalence will not change between 2012 and 2026 with the study-specific prevalence slightly increasing for HRS and decreasing for ELSA and H2000. The projections for 2014–2026 will maintain the observed study-specific trends in 2000–2012. The uncertainty in projected prevalence will increase over time, as shown by the 95% credible intervals.

**Table 3:** Means and 95% credible intervals of differences of projected prevalences between 2012 and 2026, and between scenarios pairwise in 2026

Outcome	Study^a^	2026 vs. 2012: Scenario 0	2026: Scenario 2 vs. Scenario 0	2026: Scenario 1 vs. Scenario 0	2026: Scenario 2 vs. Scenario 1
**Stair climbing limitations**	ALL	0.7 (− 4.0, 6.5)	− 9.6 (− 15.6, − 3.9)	−8.2 (− 13.5, − 3.6)	− 1.4 (− 6.3, 3.9)
	HRS	5.4 (0.0, 11.6)	−10.9 (− 17.7, − 4.3)	− 9.1 (− 15.5, − 3.8)	−1.7 (− 6.7, 2.7)
	ELSA	−2.6 (− 8.1, 3.5)	− 8.7 (− 15.1, − 3.3)	−7.5 (− 12.9, − 2.0)	−1.1 (− 5.7, 3.8)
	H2000	−3.3 (− 9.1, 3.4)	− 9.0 (− 15.6, − 2.4)	− 7.6 (− 13.4, − 0.9)	−1.4 (− 6.4, 5.2)
**Walking limitations**	ALL	−2.7 (−4.8, 0.3)	− 1.9 (− 4.6, 0.2)	−0.7 (− 3.4, 1.9)	−1.3 (− 3.5, 0.5)
	HRS	−3.0 (− 5.5, 0.8)	− 2.1 (− 5.4, 0.4)	−0.8 (− 4.3, 2.7)	−1.3 (− 3.9, 0.9)
	ELSA	−2.2 (−4.5, 0.9)	− 1.8 (− 5.1, 0.2)	−0.6 (− 3.4, 1.7)	−1.2 (− 3.4, 0.8)
	H2000	−3.7 (− 6.5, − 0.1)	− 1.7 (− 4.4, 0.5)	− 0.5 (− 3.3, 2.1)	−1.3 (− 4.4, 0.9)

^a^*HRS* The Health and Retirement Study, *ELSA* The English Longitudinal Study of Ageing, *H2000* The Health 2000 and 2011 Surveys

#### Projected prevalences between scenarios

The prevalence of stair climbing limitations will be lower in scenarios 1 and 2 compared to scenario 0 with the prevalence in 2026 29.1% (CrI 24.4, 35.2%) for scenario 0, 20.9% (CrI 17.3, 26.0%) for scenario 1, and 19.5% (CrI 14.7, 24.7%) for scenario 2 (Fig. 2, Table 3). All the study-specific prevalences will also be lower for scenarios 1 and 2 compared to scenario 0 in 2016. For walking limitations, there will be no differences between the prevalence estimates under different scenarios.

## Discussion

Our projections suggest that the prevalence of walking limitations will decrease between 2012 and 2026 while there will be no change in the prevalence of stair climbing limitations. Other studies have reported an increase in mobility limitations in the future [23, 59]. Our results may be due to the selection proportion of variable study being lower for walking limitations compared to stair climbing limitations (Table 2), which in turn caused the study-specific projections to develop similarly to each other (Fig. 2, Table 3). When variable study had a high selection proportion, the study-specific projections maintained the corresponding trends of observed data.

Our results also indicate that implementing a physical activity intervention in the older population in 2014 would decrease the projected prevalence of stair climbing limitations from 2014 to 2026 compared with the projection with no intervention. The projected prevalence was about 10% lower in 2026 if a physical intervention would have been carried out in the population compared with no intervention. For walking limitations, the physical activity intervention did not appear to influence projected prevalence. For stair climbing limitations, ADL and IADL score were selected as predictors (Table 2), which is necessary for the intervention to influence projected prevalence of outcomes. However, the intervention did not affect the projected prevalence of walking limitations, despite ADL and IADL score being predictors. This may be contributed by the fact that low prevalence of walking limitations in the observed data was decreasing in projections without the intervention effect (scenario 0).

Physical activity interventions have been recommended as they reduce functional limitations in the society [15, 16, 60]. Our results suggest that a physical activity intervention in 2014 will decrease the prevalence of stair climbing limitations in 2026. Since only individuals aged 76 years or older were included in our results the effect of the intervention might be underestimated compared to analysing younger age groups [60].

Our study has several strengths. Our method is a non-parametric method automatically incorporating possible interactions, nonlinearities and different lags of predictors. The construction of parametric models for both the outcomes and risk factors while accounting for these complications can be time-consuming whereas the machine-learning (tree-based methods) methods can avoid these. The use of longitudinal data allows us to exploit the individual-level transitions in predictors and mobility. Also, our method takes into account different sources of uncertainty. We utilised the ATHLOS dataset which provided harmonised data from different countries. The baseline and follow-up participation rates were mostly high across studies, which reduces the potential bias due to non-participation.

Our study has some limitations. We incorporated the result of a meta-analysis by Roberts et al. [29] using SMD approach as alternative scenarios into our projections. The problem with SMD approach is that it entices to include studies with small SDs leading to high SMDs and overestimated results. We implemented the intervention effect at 2014 and assumed the effect to remain thereafter. Also, the physical activity interventions included in the meta-analysis might not be feasible at population level. In alternative scenarios defined in our analysis the intervention effect was added to only those with no vigorous physical activity, since only vigorous physical activity was available in HRS, ELSA, and H2000 in ATHLOS. This may cause overestimation in the results, since in the meta-analysis by Roberts et al. [29] interventions with moderate physical activity were included. Also, our method requires data with equal intervals between repeated measures which influences the general usability of the method. We excluded the refreshment samples from our analysis dataset limiting how far into the future we can generate projections, in our case until 2026. Furthermore, since our method is based on observational data, the relationship between predictors and outcomes is not necessarily causal.

## Conclusions

Implementing a physical activity intervention in older population appears to have a positive effect on mobility limitations in the future. Our method presents a novel approach to generate projections utilizing longitudinal survey data consisting of several cohorts with the possibility to include scenarios. For future considerations, our method should be compared against other projection methods.

## Supplementary Information

**Additional file 1: Supplementary Material.** Search strategy of the literature review, description of other functional limitation outcomes, description of the preparation of data and imputation process, theory behind generating projections, additional analysis, and R-code for generating projections.

## Data Availability

The datasets generated and/or analysed during the current study are available in the ATHLOS repository, https://athlos.pssjd.org/ [61].

## Abbreviations

ATHLOS: Ageing Trajectories of Health – Longitudinal Opportunities and Synergies
ADL: Activities of daily living
IADL: Instrumental activities of daily living
RCT: Randomised controlled trial
SMD: Standardised mean difference
CI: Confidence interval
HRS: The Health and Retirement Study
ELSA: The English Longitudinal Study of Ageing
H2000: The Health 2000 and Health 2011 Surveys
NDI: National Death Index
NHS: National Health Service
MI: Multiple imputation
CART: Classification and regression trees
SD: Standard deviation
CrI: Credible interval
