# Supplementary material for: Using multiple imputation and intervention-based scenarios to project the mobility of older adults
Source: BMC Geriatr. 2022 Apr 9;22:311. doi: 10.1186/s12877-022-03008-4 (PMC8994920; doi:10.1186/s12877-022-03008-4)
Supplement: Supplementary file 1 — Additional file 1: Supplementary Material. Search strategy of the literature review, description of other functional limitation outcomes, description of the preparation of data and imputation process, theory behind generating projections, additional analysis, and R-code for generating projections. [file 12877_2022_3008_MOESM1_ESM.pdf]

Supplementary Material

Kontto J, Paalanen L, Sund R, Sainio P, Koskinen S, Demakakos P, Tolonen H, Härkänen T:

Using multiple imputation and intervention-based scenarios to project the mobility of older adults

Contents

- Literature review on RCT results: search strategy 2
- Other functional limitation outcomes: vision, memory, and hearing limitations 3
- The preparation of data and imputation of missing values 4
    - Supplementary Fig. S1a–e 5
- Generating projections – theory 10
- Results for vision, memory, and hearing limitations 11
    - Supplementary Table S1 12
    - Supplementary Fig. S2 13
    - Supplementary Table S2 14
- Transition probabilities between 2010 and 2012 15
    - Supplementary Table S3 16
- R-code for generating projections 17
- References 25

Literature review on RCT results: search strategy

Medline OVID 18 April 2019

1 "*Aged, 80 and over"/ or *Aged/ (17151)

2 (elderly or ageing or aging or "old population*" or "older population* older citizen*" or "older adult*").ti. (204750)

3 1 or 2 (213723)

4 "Systematic Review"/ (104632)

5 (systematic review or meta-analysis).pt. (161843)

6 4 or 5 (161843)

7 "Activities of Daily Living"/ or "Quality of life"/ or "mental competency"/ or cognition/ or autonomy/ or "independent living"/ or exp "Health Behavior"/ or "Health Risk Behavior"/ or Risk Reduction Behavior/ or "Physical Fitness"/ or Muscle Strength/ or "Health status"/ or "social capital"/ or "International Classification of Functioning, Disability and Health"/ or "Community Participation"/ or "Social Participation"/ or exp Health Behavior/ or Personal Satisfaction/ or Social Networking/ or Sedentary behavior/ or Adaptation, Psychological/ or Social Support/ or Mental Health/ or Lifestyle/ or Healthy Lifestyle/ (876298)

8 (function* or cognitive or cognition or independen* or autonomy or mobility or "physical activity" or "physical health" or "health status" or "muscle strength" or fitness or inactiv* or sedentary or "successful ageing" or "successful aging" or "healthy aging" or "healthy ageing" or "healthy living" or "health habits" or "quality of life" or "activities of daily living" or "social capacity" or "health behavi*r" or engag* or participat* or "social networks" or "social life" or satisfactory or satisfaction ot coping or capability or capacity or psychosocial or "social network*" or "risk reduction" or lifestyle).ti. (1167801)

9 7 or 8 (1851444)

10 (intervention* or therap* or treatment* or treated or program* or policy or policies or tool* or device* or technique* or training or coaching or counsel* or education or prevention or preventive or "health promotion" or "clinical trial*" or "clinical stud*" or rct* or random*).ti,ab,sh. (10385025)

11 (benefit* or promot* or effect* or efficacy or impact* or outcome* or improve* or increas* or decreas*).ti. (3167893)

12 10 or 11 (11972623)

13 3 and 6 and 9 and 12 (597)

The search yielded 597 results. After crude selection by the information specialist 172 articles remained. Of these, 16 articles with outcome related to physical functioning were selected for further evaluation based on the estimated applicability to ATHLOS. For example, reviews with too specific interventions such as yoga or aquatic exercise were excluded, as well as reviews including interventions amongst a homogenous study population such as only obese participants.

Other functional limitation outcomes: vision, memory, and hearing limitations

In our study, we selected outcomes representing different dimensions of functional ability. In order to define one set of dimensions, we utilized the results by Caballero et al. [1] who developed a health metric in the ATHLOS project. In their procedure, 45 items related to functioning, and available in the ELSA baseline data, were assessed with exploratory factor analysis. As a result, five factors were identified representing the following dimensions of functioning: mobility, eyesight, cognitive functioning, psychological functioning, and hearing. In addition to two items from mobility factor, we selected an item with the highest loading from each other dimensions of functioning as outcomes with one precondition: the item is available in all three studies (HRS, ELSA, H2000). The highest loading of items of the psychological functioning factor was 0.54 so no outcome was selected from that factor. As a result, three functional limitations were selected as outcomes: 1) Limitations in near vision after correction with glasses; 2) Limitations in delayed recall; and 3) Limitations in hearing. The selected outcomes were based on self-reported information, except memory limitations, where delayed recall test with ten words was used. Those who recalled less than five words out of ten immediately after three rounds of learning the words were defined as having limitations in short-term memory [2].

The preparation of data and imputation of missing values

A bootstrap sample is in wide format, in which all observed data of both the outcomes and predictors of an individual are on the same row, and denoted by $X = (X_1, \ldots, X_t)$, where waves $1, \ldots, t$ correspond the years 2000–2012 biennially. We simplify the notation by omitting indices relating to individuals. In order to have compatible years across studies, the participants of the Health 2011 Survey were randomly assigned into 2010 and 2012.

Multiple imputation based on chained equations and classification and regression tree (CART) [3, 4] was implemented to impute the missing values of X using the package 'mice' of the R software [5]. Since the number of variables in X was 183 (26 variables across 7 waves in addition to time-independent variable study), the complexity parameter value (cp) in function 'rpart' was set to 0.001 and the minimum number of observations in any terminal node (minbucket) was set to 100 to reduce the number of predictors in the imputation models for each variable [6]. For each full conditional imputation model all variables, which were selected in the CART model, were included. Each variable can depend on other variables measured at the same time, and all variables at the previous or following (if observed) waves. Age, sex, and education for 2012 are forced as predictors to all imputation models. Then, the missing values of X were imputed with function 'mice' where cp was set to 0.0001 and minbucket set to 25.

Some of the observed prevalence of Health2000-11 outcomes differed from the corresponding prevalence in other studies. Also, the imputed prevalence for 2002–2008 in Health2000-11 was strongly influenced by the lower prevalence in HRS and ELSA (Supplementary Fig. S1a–e). Thus, Health2000-11 outcomes of 2002–2008 are re-imputed together with the HRS data using generalised linear mixed models with the logit link and the Markov Chain Monte Carlo (MCMC) method [7]. For this purpose, this subset of the data is converted to a long format with age, sex, education, mortality status, and outcomes of 2000–2010 included. Outcome variables were imputed stepwise. First, the missing values of the first outcome of 2002–2008 were imputed with age, sex, education, and mortality status as predictors. Then, the missing values of the second outcome are imputed with also the first outcome as a predictor. This is repeated until all outcomes of 2002–2008 are imputed. The imputed data are then transformed back to the wide format and merged with the other data.

Supplementary Fig. S1a–e. Imputed datasets across bootstrap samples by outcome. Imputed values for the Health 2000 and 2011 Surveys are included both with and without using generalized linear mixed models with the logit link and the Markov Chain Monte Carlo (MCMC) method. Only individuals aged 76 years or older at each year are included.

Supplementary Fig. S1a. Stair climbing limitations

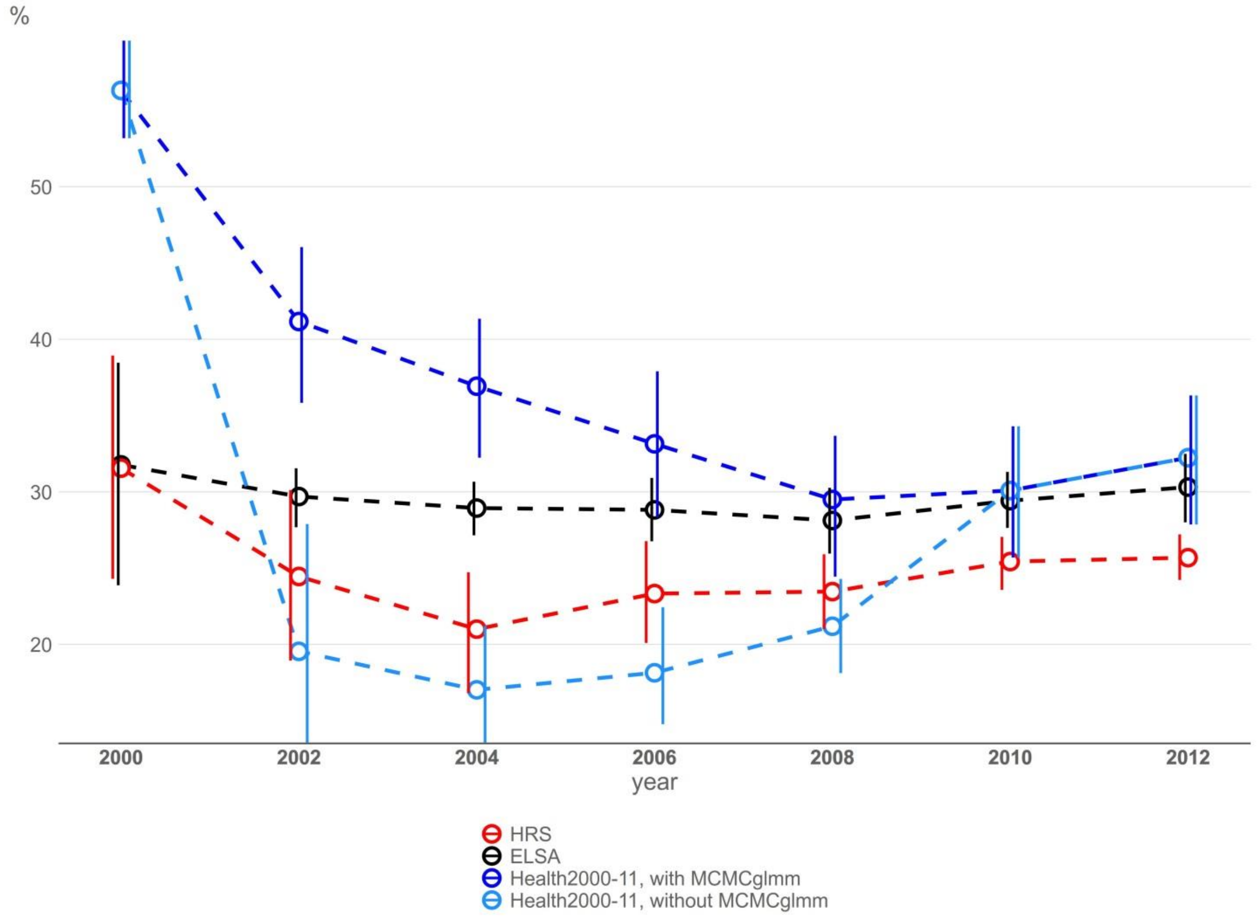

Supplementary Fig. S1b. Moving limitations

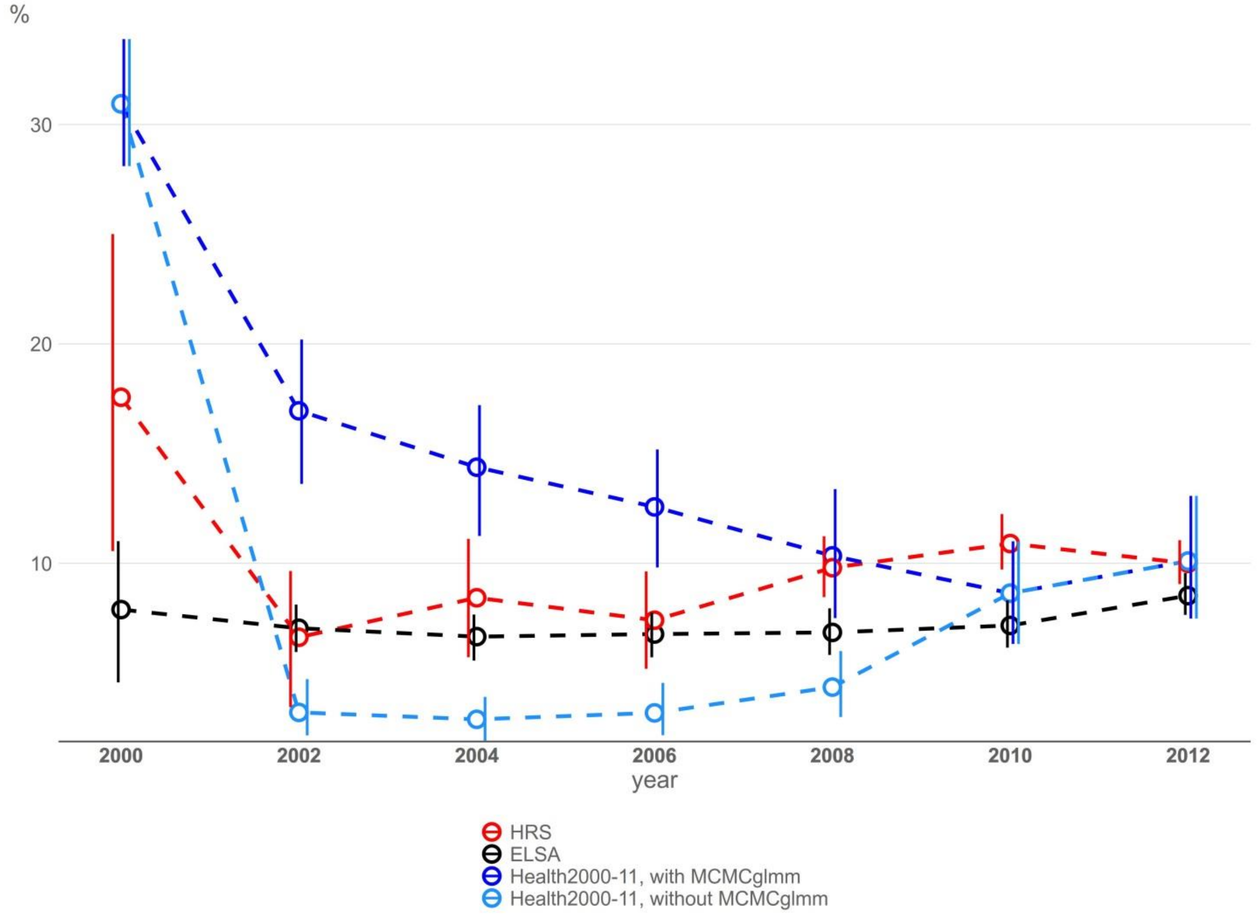

Supplementary Fig. S1c. Vision limitations

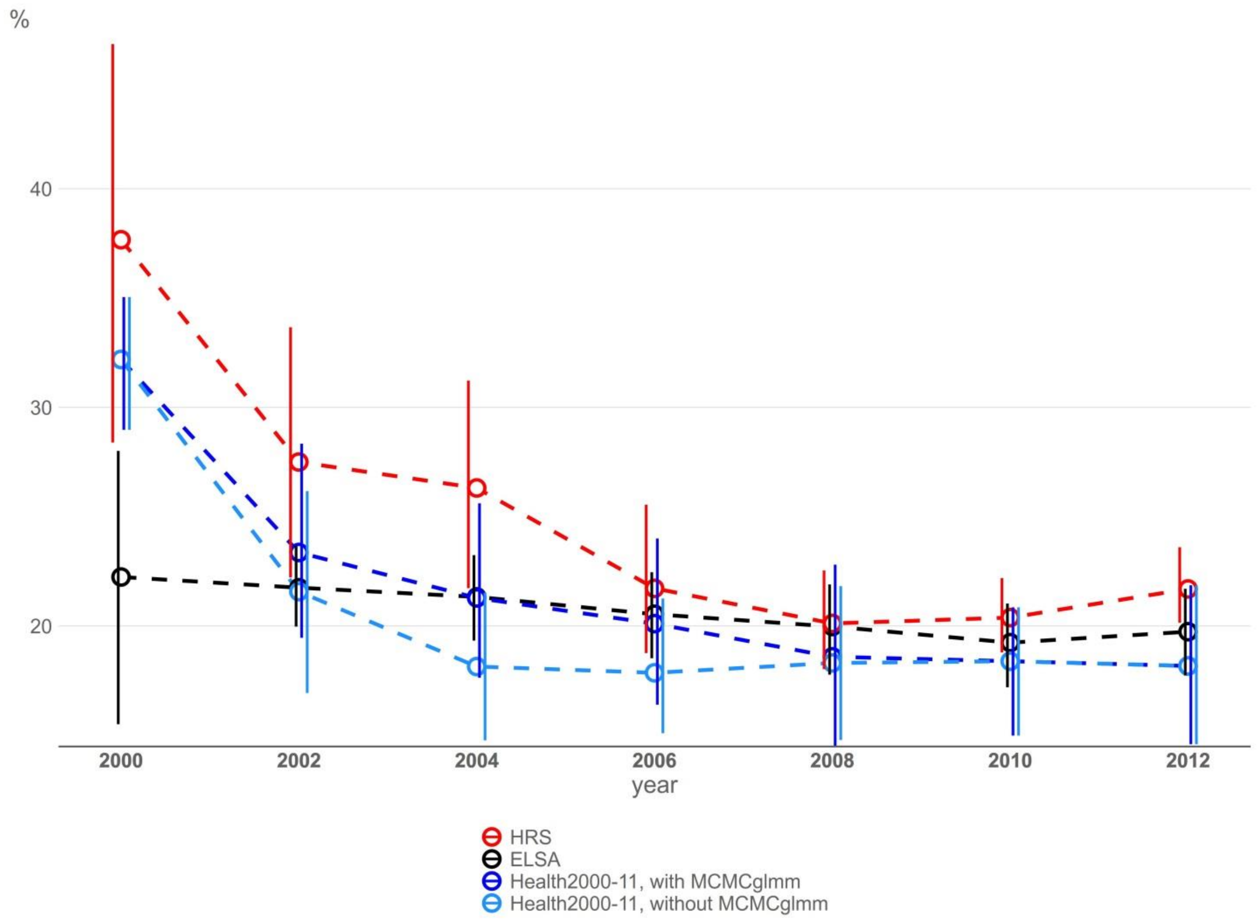

Supplementary Fig. S1d. Memory limitations

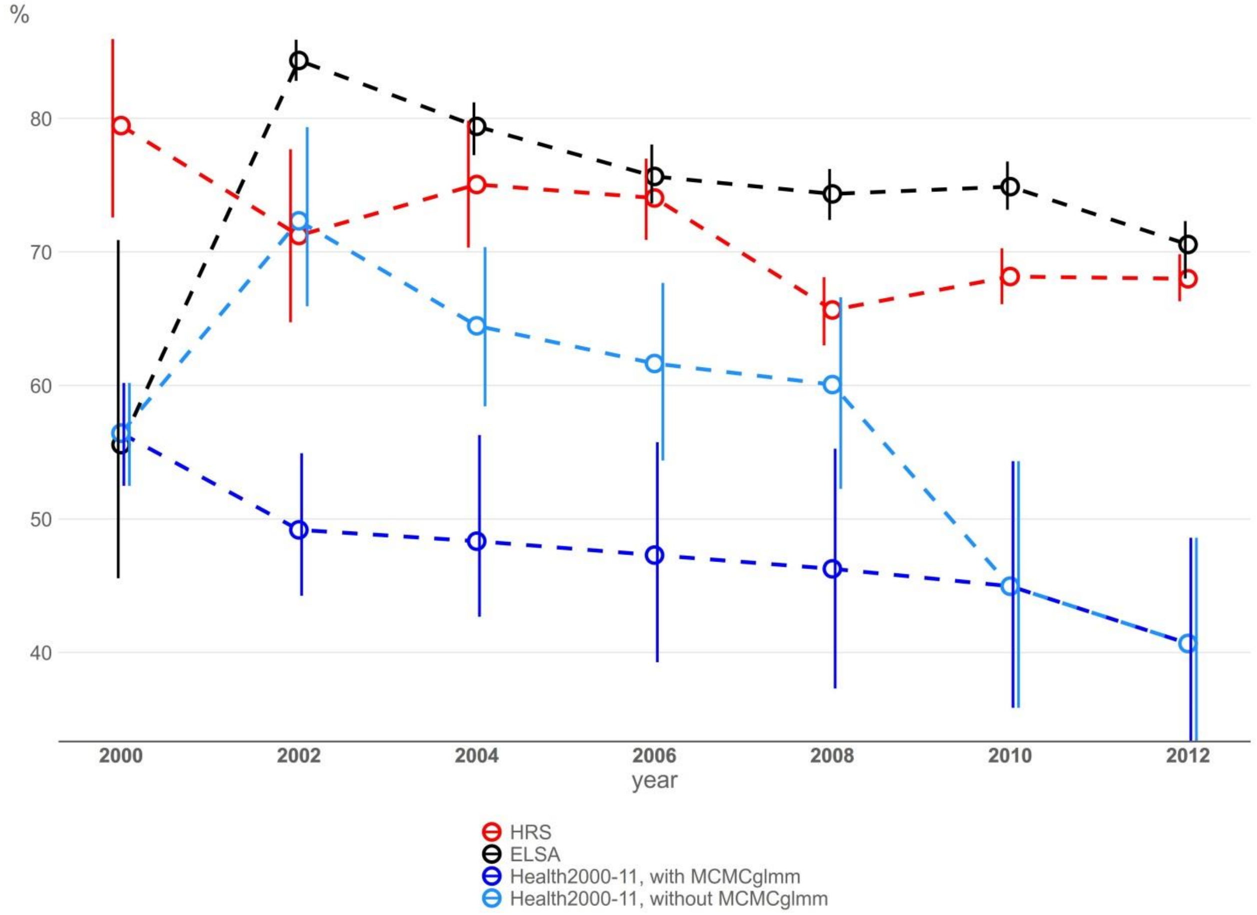

Supplementary Fig. S1e. Hearing limitations

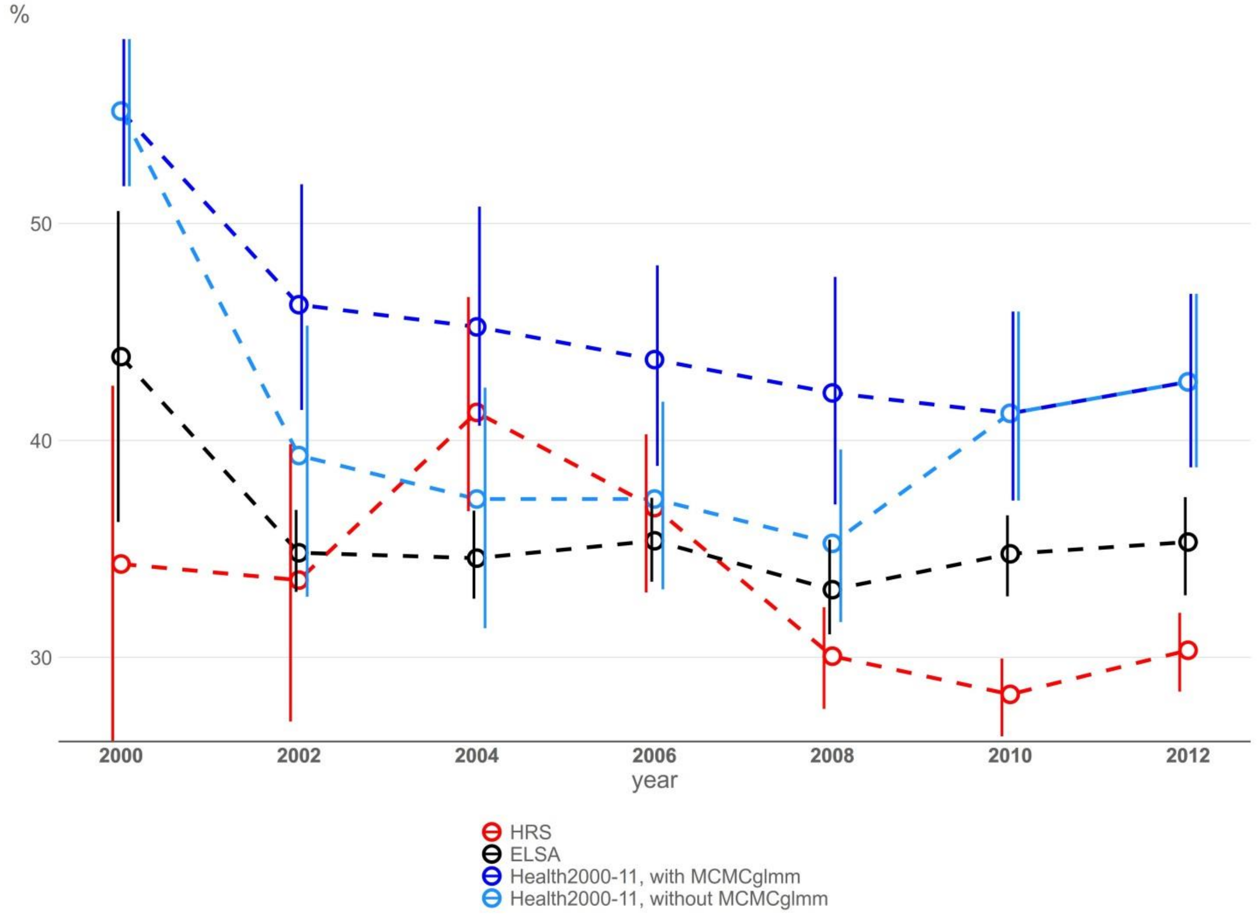

Generating projections – theory

Formally, the projections of I^{t+1} were assumed to depend on $I^1, \dots, I^t$. The conditional distributions $P(I^{t+1}|I^1, \dots, I^t)$ for $t+1, \dots, t+s$ allowing us to produce the joint distribution of the variables in the future time-points sequentially using the chain rule:

$$P(I^{t+1},\dots,I^{t+s}|I^1,\dots,I^t) = P(I^{t+1}|I^1,\dots,I^t)P(I^{t+2}|I^1,\dots,I^{t+1})\dots P(I^{t+s}|I^1,\dots,I^{t+s-1}).$$

Here, we assumed that the projections for wave $t+s$ could depend only on $I^s,\dots,I^{t+s-1}$ as I consisted of t waves:

$$P(I^{t+s}|I^1,\dots,I^{t+s-1}) = P(I^{t+s}|I^s,\dots,I^{t+s-1}).$$

These distributions could be formulated using the predictive distributions of Bayesian inference:

$$P(I^{t+1}|I^1,\dots,I^t) = \int P(I^{t+1}|\theta, I^1,\dots,I^t)\, dP(\theta|I^1,\dots,I^t).$$

$$P(I^{t+s}|I^1,\dots,I^t) = \int \cdots \int P(I^{t+1},\dots,I^{t+s}|\theta, I^1,\dots,I^t)\, dI^{t+1} \cdots\, dI^{t+s-1} dP(\theta|I^1,\dots,I^t).$$

The imputed values approximated the Bayesian predictive distributions which is a proper imputation method, as the different sources of uncertainty (parameter, model and prediction uncertainties) are accounted for [8, 9]. CART can adapt to possible interactions and non-linearities of the variables.

Results for vision, memory, and hearing limitations

Selection of predictors

For vision, memory, and hearing limitations all previous measurements were among selected predictors (Supplementary Table S1). IADL score in 2012 (48%) and ADL score in 2012 (13%) were predictors for vision limitations, and IADL score in 2012 (19%) for memory limitations. Some of IADL and ADL scores from 2000–2010 were also predictors for vision, memory, and hearing, but the selection proportions were low, varying between 11% and 29%. Time-independent variable study was a predictor for all outcomes. The selection proportion varied between 42% and 100%. The predictors of vision limitations were concentrated on 2010–2012, whereas the predictors of memory and hearing limitations were more evenly distributed to all years. No outcomes had predictors in 2012 with selection proportion at least 50%.

Projected prevalence until 2026 under scenario 0

The projections from 2014 to 2026 will maintain the observed study-specific trends in 2000 to 2012 (Supplementary Fig. S2, Supplementary Table S2). The uncertainty in projected prevalence will increase over time, as shown by the 95% credible intervals. The change of prevalence will go to opposite directions in HRS and H2000 for hearing limitations. In HRS, the prevalence will increase from 30.3% (CI 28.3%, 31.9%) to 36.7% (CrI 29.3%, 42.9%) while there will be a decrease in H2000 (42.8% [CI 39.4%, 47.2%] vs. 34.0% [CrI 26.9%, 42.4%]). For memory limitations, the prevalence will slightly increase in HRS and decreased in other studies. There will be no change in the prevalence of vision limitations.

Projected prevalences until 2026 between scenarios

For all outcomes, there will be no differences between the prevalence estimates under different scenarios.

Supplementary Table S1. The predictors of outcomes in 2012 by survey year. Numbers in parentheses are the proportions of bootstrap samples in which a variable was selected as a predictor. Variables with selection proportions at least 10% are included in the table. Age, sex, and education in 2012 are forced as predictors to all imputation models.

	Outcome in 2012		
Predictors[a]	Vision limitations	Memory limitations	Hearing limitations
**2012**	IADL (48)<br>climbing (39)<br>hearing (38)<br>mortality (24)<br>alcohol (22)<br>ADL (13)	alcohol (38)<br>IADL (19)<br>climbing (14)<br>mortality (11)<br>hearing (11)	vision (45)
**2010**	vision (100)<br>IADL (29)<br>education (17)<br>mortality (11)	memory (100)<br>IADL (27)<br>education (16)<br>mortality (11)	hearing (100)<br>education (18)<br>VPA (13)
**2008**	vision (98)	memory (100)<br>IADL (18)<br>alcohol (12)<br>education (11)	hearing (99)<br>education (14)<br>VPA (10)
**2006**	vision (16)<br>education (12)	memory (72)<br>VPA (12)<br>IADL (11)	hearing (66)<br>memory (14)<br>VPA (13)<br>education (11)<br>ADL (11)
**2004**	vision (16)	memory (100)<br>alcohol (34)	hearing (92)<br>memory (12)
**2002**	vision (26)	memory (61)<br>alcohol (23)<br>VPA (12)	hearing (31)<br>vision (12)<br>VPA (12)
**2000**	vision (29)<br>IADL (12)<br>education (11)	memory (52)<br>education (29)<br>alcohol (15)<br>IADL (12)	hearing (25)<br>education (18)
**time-independent**	study (42)	study (100)	study (100)

[a] IADL = IADL score; ADL = ADL score; alcohol = alcohol use; VPA = vigorous physical activity; climbing = stair climbing limitations; moving = moving limitations; vision = vision limitations; memory = memory limitations; hearing = hearing limitations; mortality = mortality status

Supplementary Fig. S2. Projections until 2026 by scenario and study

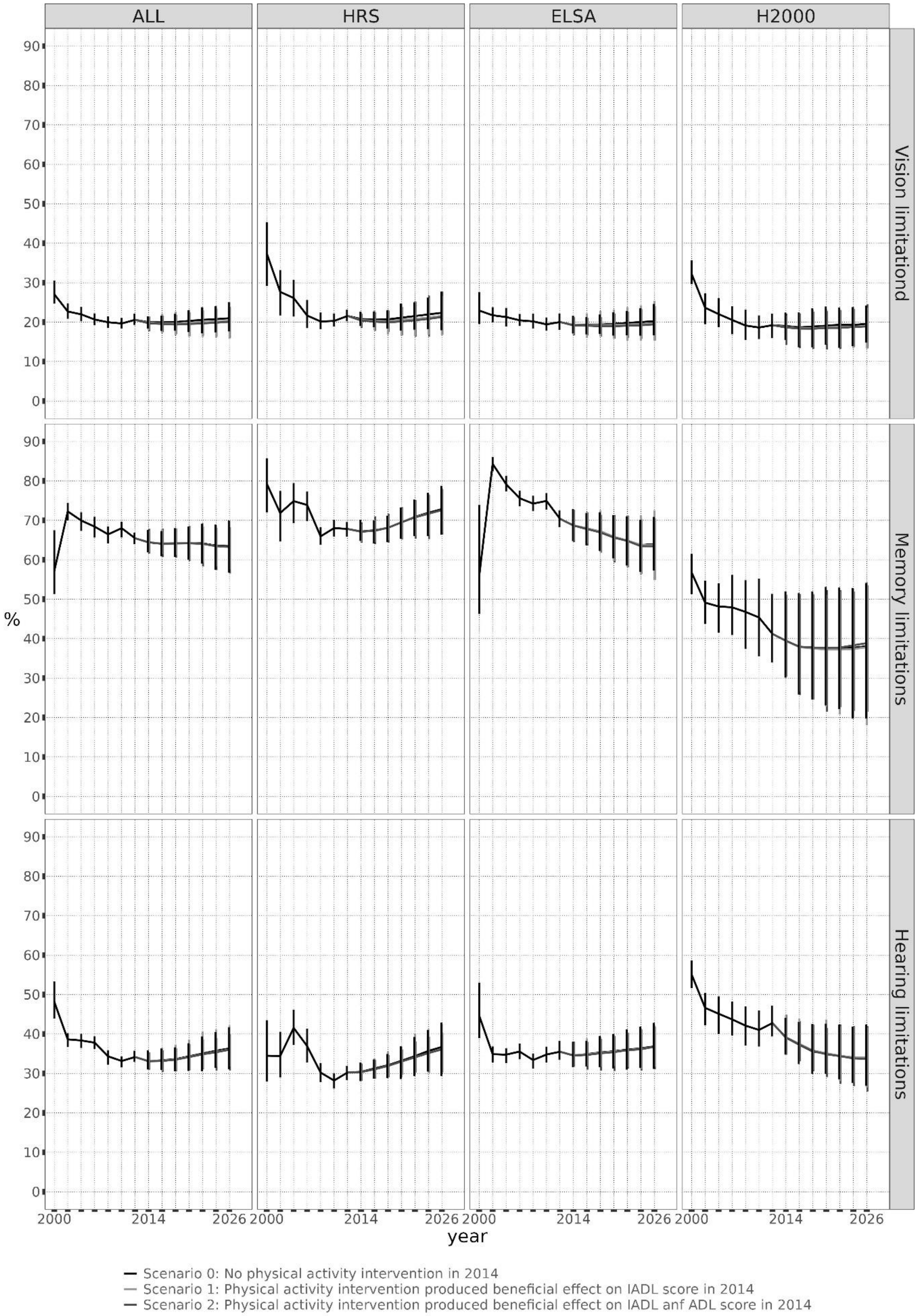

Supplementary Table S2. Means and 95% credible intervals of differences of projected prevalences between 2012 and 2026, and between scenarios pairwise in 2026.

Outcome	Study[a]	2026 vs. 2012: Scenario 0	2026: Scenario 2 vs. Scenario 0	2026: Scenario 1 vs. Scenario 0	2026: Scenario 2 vs. Scenario 1
**Vision limitations**	ALL	0.4 (-2.9, 4.0)	-0.9 (-5.1, 4.3)	-0.7 (-5.4, 4.1)	-0.2 (-4.6, 4.0)
	HRS	0.8 (-3.5, 5.3)	-1.1 (-6.9, 5.1)	-0.8 (-7.3, 5.2)	-0.3 (-6.0, 6.3)
	ELSA	0.2 (-3.4, 4.0)	-0.8 (-5.7, 4.6)	-0.5 (-5.5, 4.1)	-0.3 (-4.4, 4.2)
	H2000	0.4 (-5.0, 5.5)	-0.7 (-6.1, 5.0)	-0.7 (-6.5, 5.7)	0.0 (-5.0, 5.5)
**Memory limitations**	ALL	-2.0 (-8.1, 4.2)	-0.2 (-5.7, 3.7)	-0.2 (-4.8, 4.5)	0.0 (-5.4, 4.9)
	HRS	5.0 (-1.5, 10.5)	-0.2 (-6.7, 6.0)	-0.3 (-5.7, 5.3)	0.0 (-6.7, 5.3)
	ELSA	-6.4 (-12.8, 0.4)	-0.6 (-8.5, 5.0)	-0.2 (-6.3, 5.7)	-0.3 (-6.7, 7.0)
	H2000	-3.1 (-18.1, 11.9)	0.8 (-10.5, 14.4)	-0.2 (-15.3, 13.4)	1.0 (-11.7, 12.6)
**Hearing limitations**	ALL	2.2 (-2.4, 7.3)	-0.3 (-6.0, 4.8)	-0.2 (-5.1, 5.4)	-0.1 (-5.4, 5.0)
	HRS	6.5 (0.0, 13.6)	-0.6 (-6.8, 5.9)	-0.5 (-6.1, 5.8)	-0.2 (-7.2, 5.0)
	ELSA	1.3 (-3.8, 6.4)	-0.1 (-5.4, 5.9)	-0.2 (-6.7, 5.7)	0.1 (-6.1, 6.8)
	H2000	-8.8 (-16.9, -0.1)	-0.3 (-9.8, 9.5)	0.0 (-8.1, 9.9)	-0.3 (-9.7, 9.1)

Transition probabilities between 2010 and 2012

Since the most important predictor of all outcomes was the previous measurements of the corresponding outcome, we present the transition probabilities of outcomes between 2010 and 2012. In general, the transition probabilities in HRS and ELSA were similar, whereas several probabilities of H2000 differed from the probabilities of the two other studies (Supplementary Table S3).

Difficulties in climbing one flight of stairs

Mortality in 2012 was higher among those with difficulties in climbing one flight of stairs in 2010 compared with those without difficulties in 2010 in HRS and ELSA, whereas probability to recover, and probability to deteriorate were similar across studies. Among those who survived until 2012 almost a third recovered from difficulties in climbing one flight of stairs.

Difficulties in moving around the home

Mortality in 2012 among those with difficulties in moving around the home in 2010 compared to those without difficulties in 2010 was four-fold in HRS and almost three-fold in ELSA, whereas in H2000 the mortality was equal. Among those H2000 participants who had difficulties in 2010, half of those who survived until 2012 recovered. In HRS and ELSA, the corresponding probabilities were 36% and 40% respectively.

Difficulties in memory

Mortality in 2012 was higher among those with difficulties in memory in 2010 compared with those without difficulties in 2010. Only 14% of those H2000 participants who did not have difficulties in 2010 developed difficulties in 2012, whereas in HRS and ELSA the probabilities were 44% and 43%, respectively.

Difficulties in vision or hearing

The probabilities were similar across studies. Among those who survived until 2012, the probability of those who recovered from difficulties in vision varied between 39% and 43% across studies, while 11% to 14% developed difficulties in vision. Correspondingly for difficulties in hearing, 23% to 29% recovered and 16% to 20% developed difficulties across studies.

Supplementary Table S3. Transition probabilities (%) from one outcome category in 2010 to the other category or to death in 2012. Only individuals aged 76 or older at 2010 are included.

		2010		Presence of limitations in 2012		
Outcome	Study	Presence of limitations	n	No	Yes	Dead
**Stair climbing limitations**	HRS	No	1,705	79.7	14.6	5.7
		Yes	581	25.0	56.8	18.2
	ELSA	No	1,988	72.0	16.7	11.3
		Yes	814	21.5	56.7	21.8
	H2000	No	605	72.6	16.1	11.3
		Yes	252	23.0	64.7	12.4
**Moving limitations**	HRS	No	2,038	87.6	5.6	6.9
		Yes	248	27.7	46.9	25.4
	ELSA	No	2,604	81.2	5.9	12.9
		Yes	199	25.9	40.0	34.1
	H2000	No	785	81.9	6.6	11.5
		Yes	72	41.9	45.7	12.4
**Vision limitations**	HRS	No	1,822	79.2	12.5	8.3
		Yes	464	35.2	53.8	11.0
	ELSA	No	2,258	75.4	10.8	13.8
		Yes	544	32.6	50.5	16.9
	H2000	No	697	78.4	10.5	11.1
		Yes	160	34.0	52.3	13.8
**Memory limitations**	HRS	No	731	51.0	43.6	5.5
		Yes	1,555	16.3	73.3	10.4
	ELSA	No	703	47.4	42.3	10.3
		Yes	2,099	14.2	70.0	15.8
	H2000	No	468	72.8	16.7	10.5
		Yes	389	22.3	64.8	12.9
**Hearing limitations**	HRS	No	1,641	76.8	14.7	8.5
		Yes	645	26.1	64.0	9.8
	ELSA	No	1,827	69.3	17.7	13.0
		Yes	975	22.9	60.1	16.9
	H2000	No	505	71.6	17.5	10.9
		Yes	352	19.5	67.9	12.6

R-code for generating projections

```
rbind.bs <- function(svyrep,excl.v=NULL,excl.v2=NULL,pred.v=NULL,drop.v=NULL,
                     incl.v=NULL, incl.v2=NULL,incl.v.wave=NULL, outcome.v=NULL,
                     pred.steps=(n.waves+1),scenario0=FALSE,scenario1=FALSE,
                     scenario2=FALSE,mb=25,cpm=.0001,mb.pred=200,
                     cpm.pred=0.001){
  if(scenario0) res0 <- list()
  if(scenario1) res1 <- list()
  if(scenario2) res2 <- list()
  predm1 <- list()
  imp0 <- list()
  bs <- svyrep$repweights
  tmp0 <- transf.fn2(svyrep$variables, n.waves, years.between.waves,
                     education=TRUE)
  rownames(tmp0) <- NULL
  #add variable step:
  tmp0$step <- 1
  ## Set variables with a small number of possible values as factor variables:
  f.v <- c()
  for (i in which(sapply(tmp0, function(x) is.numeric(x) &&
                         length(unique(x)) <= 5))) {
    tmp0[,i] <- factor(tmp0[,i])
    f.v <- c(f.v, i)
  }
  ## Set character variables as factor variables:
  for (i in which(sapply(tmp0, function(x) is.character(x))))
    tmp0[,i] <- factor(tmp0[,i])

  for (i.b in 1:ncol(bs)) {
    tmp.all <- tmp0[rand.rep(bs[,i.b]),]
    rownames(tmp.all) <- NULL

    #split h2011 individuals randomly into waves 6 and 7:
    nobs <- sum(tmp.all$study=='Health2000-2011')
    nrand <- (runif(n=nobs))<0.5
    mov.vars <- names(tmp.all)[grepl("\\.7$", names(tmp.all))]
    mov.vars <- gsub('.7','',mov.vars)
    mov.vars <- mov.vars[!mov.vars%in%c('entry_wave','age','sex')]
    tmp.all[tmp.all$study=='Health2000-2011',paste0(mov.vars,'.7')][nrand,] <-
      tmp.all[tmp.all$study=='Health2000-2011',paste0(mov.vars,'.6')][nrand,]
    tmp.all[tmp.all$study=='Health2000-2011',paste0(mov.vars,'.6')][nrand,] <-
      NA
    tmp.all$part_status.6[tmp.all$study=='Health2000-2011' &
                          is.na(tmp.all$part_status.6)] <- FALSE
    tmp.all$living_status.6[tmp.all$study=='Health2000-2011' &
                            is.na(tmp.all$living_status.6) &
                            tmp.all$living_status.7=='Alive'] <- 'Alive'
    tmp.all$living_status.7[tmp.all$study=='Health2000-2011' &
                            is.na(tmp.all$living_status.7) &
                            tmp.all$living_status.6=='Dead'] <- 'Dead'

    for (i.pred in 1:pred.steps){
      if(i.pred==1){
        tmp <- tmp.all
        #selecting predictors and imputing observed data:
        pred.m <- matrix(0, ncol(tmp), ncol(tmp)) #all zeros
        dimnames(pred.m) <- list(colnames(tmp), colnames(tmp))
        for (i in 1:ncol(tmp)) {
          if(!colnames(tmp)[i]%in%c(excl.v,pred.v,excl.v2)){
            if(length(unique(setdiff(tmp[,colnames(tmp)[i]], NA))) > 1) {
```

```
      tmp.m <- my.eval("rpart(", colnames(tmp)[i], " ~ ",
                       paste0(setdiff(
                         colnames(tmp)[-i],
                         c(excl.v,excl.v2)), collapse="+"),
                       ", data=tmp, ",
                       "control=rpart.control(minbucket=mb.pred,
                       cp=cpm.pred))")
      if(is.factor(tmp$frame$var))
        pred.m[i,] <- as.numeric(colnames(tmp) %in%
                                 levels(tmp.m$frame$var))
      else pred.m[i,] <- as.numeric(colnames(tmp) %in% tmp.m$frame$var)
}}}
pred.m[!rownames(pred.m) %in% c(excl.v, pred.v, excl.v2),
       colnames(pred.m) %in% incl.v] <- 1
if(!is.null(incl.v2))
  pred.m[!rownames(pred.m) %in% c(excl.v, pred.v, excl.v2),
         colnames(pred.m) %in% incl.v2] <- 1

diag(pred.m) <- 0

imp.data <- is.na(tmp)
#this includes both adl_score and iadl_score:
imp.data[,grep('adl_score',names(tmp))] <- FALSE

## Sample predicted values:
tmp.mi <- mice(tmp, m=1, maxit=10, predictorMatrix=pred.m,
               where=imp.data, method="cart", minbucket=mb, cp=cpm,
               remove.collinear=FALSE, printFlag=FALSE)
tmp <- mice::complete(tmp.mi, 1)

for(i in 1:n.waves){
  adl.vars <- c('incontinence.','adl_toilet.','adl_bathing.',
               'adl_dressing.','adl_eating.','adl_bed.')
  iadl.vars <- c('iadl_phone.','iadl_shopping.','iadl_meals.',
                'iadl_housewk.','iadl_medication.','iadl_money.')
  adl.score <- apply(tmp[,paste0(adl.vars,i)],1,function(a)
    sum(a=='Absence of difficulties'))
  iadl.score <- apply(tmp[,paste0(iadl.vars,i)],1,function(a)
    sum(a=='Absence of difficulties'))
  tmp[[paste0('adl_score.',i)]][is.na(tmp[[paste0('adl_score.',i)]])]
  <- adl.score[is.na(tmp[[paste0('adl_score.',i)]])]
  tmp[[paste0('iadl_score.',i)]][is.na(tmp[[paste0('iadl_score.',i)]])]
  <- iadl.score[is.na(tmp[[paste0('iadl_score.',i)]])]
}

#MCMCglmm:
tmp <- impute.mcmcglmm(tmp)

if(pred.steps>1){ #generating pred.m1 only if pred.steps>1

  #excluding adl and iadl items and
  #creating new prediction matrix for projections
  tmp <- tmp[,!names(tmp)%in%drop.v]

  pred.m1 <- matrix(0, ncol(tmp), ncol(tmp)) #all zeros
  dimnames(pred.m1) <- list(colnames(tmp), colnames(tmp))

  for (i in 1:ncol(tmp)) {
    #Done only for wave 7 variables:

    if(grepl("\\.7$", colnames(tmp)[i]) & !colnames(tmp)[i] %in%
```

```
        c(excl.v,pred.v)){
        if(length(unique(setdiff(tmp[,colnames(tmp)[i]], NA))) > 1) {
          tmp.m <- my.eval("rpart(", colnames(tmp)[i], " ~ ",
                           paste0(setdiff(colnames(tmp)[-i], c(excl.v)),
                                  collapse="+"),
                           ", data=tmp, ",
                           "control=rpart.control(minbucket=mb.pred,
                           cp=cpm.pred))")
          if(is.factor(tmp.m$frame$var))
            pred.m1[i,] <-
              as.numeric(colnames(tmp) %in% levels(tmp.m$frame$var))
          else
            pred.m1[i,] <-
              as.numeric(colnames(tmp) %in% tmp.m$frame$var)
    }}}

    pred.m1[grepl("\\.7$", rownames(pred.m1)) & !rownames(pred.m1)
           %in% c(excl.v, pred.v), colnames(pred.m1) %in% incl.v] <- 1
    diag(pred.m1) <- 0
  }

  tmp.imp <- tmp #saving data after step 1

}
## Add new rows in the dataframe as new prediction step:
if(i.pred==2){
  tmp2 <- subset(tmp, living_status.7=='Alive', select=-c(step))
  tmp2 <- tmp2[,!grepl("\\.1$", colnames(tmp2))]
  colnames(tmp2) <- unlist(lapply(
    strsplit(colnames(tmp2),'[.]'),function(a)
      ifelse(length(a)<2,a,paste0(a[1],'.',as.numeric(a[2])-1))))
  tmp <- rbind.fill(tmp, cbind(step=2, tmp2))
  rm(tmp2); gc()
  ## Make some transformations e.g. add age some constant,
  ## copy sex the same value etc
  tmp <- rbind(subset(tmp, as.numeric(step) < 2),
               transf.fn2(subset(tmp, as.numeric(step) == 2),
                          n.waves, years.between.waves,
                          education=TRUE))

  #Scenario 0:
  if(scenario0){
    tmp.mi.scenario0 <- mice(tmp, m=1, maxit=10, predictorMatrix=pred.m1,
                             method="cart", minbucket=mb, cp=cpm,
                             printFlag=FALSE)
    tmp.scenario0 <- mice::complete(tmp.mi.scenario0, 1)
    tmp.res0 <- subset(tmp.scenario0,
                       select=c('study','step',
                                colnames(tmp.scenario0)
                                [grep("\\.7$",colnames(tmp.scenario0))]))
  }

  #Scenario 1:
  if(scenario1){
    pred.m2 <- pred.m1
    #living_status and outcome(s) not imputed and not used as predictors:
    pred.m2[,paste0(c('living_status.',paste0(outcome.v,'.')),n.waves)]
    <-0
    pred.m2[paste0(c('living_status.',paste0(outcome.v,'.')),n.waves),]
    <-0
    imp.data <- is.na(tmp)
```

```
  imp.data[,paste0(c('living_status.',paste0(outcome.v,'.')),n.waves)]
  <-FALSE

  tmp.mi.scenario1 <- mice(tmp, m=1, maxit=10, predictorMatrix=pred.m2,
                           where=imp.data, method="cart", minbucket=mb,
                           cp=cpm, printFlag=FALSE)
  tmp.scenario1 <- mice::complete(tmp.mi.scenario1, 1)
  sd.hrs <-
    with(subset(tmp.scenario1,step==1&study=='HRS'),
         sd(iadl_score.1))
  sd.elsa <-
    with(subset(tmp.scenario1,step==1&study=='ELSA'),
         sd(iadl_score.2))
  sd.h2000 <-
    with(subset(tmp.scenario1,step==1&study=='Health2000-2011'),
         sd(iadl_score.1))
  #define scenario 1 (by cohort):
  sds.iadl <- c(sd.hrs, sd.elsa, sd.h2000)
  sts <- c('HRS','ELSA','Health2000-2011')
  for(i in 1:3){
    tmp.subset <- tmp.scenario1$step==2 &
      tmp.scenario1[[paste0('vig_pa.',n.waves)]]=='No' &
      tmp.scenario1$study==sts[i]
    tmp.scenario1[[paste0('iadl_score.',n.waves)]][tmp.subset] <-
      round(rnorm(length(tmp.scenario1[[paste0('iadl_score.',n.waves)]]
                         [tmp.subset]),
        mean=tmp.scenario1[[paste0('iadl_score.',n.waves)]]
        [tmp.subset]+(1.12*sds.iadl[i]), sd=sds.iadl[i]))
  }
  #restrict scenario variable values:
  tmp.scenario1[[paste0('iadl_score.',n.waves)]]
  [tmp.scenario1$step==2 &
      tmp.scenario1[[paste0('iadl_score.',n.waves)]]>6] <- 6
  tmp.scenario1[[paste0('iadl_score.',n.waves)]]
  [tmp.scenario1$step==2 &
      tmp.scenario1[[paste0('iadl_score.',n.waves)]]<0] <- 0

  pred.m2 <- pred.m1
  #pa not used as a predictor for outcome(s) and mortality:
  pred.m2[,paste0('vig_pa.',n.waves)] <- 0

  tmp.mi.scenario1 <- mice(tmp.scenario1, m=1, maxit=10,
                           predictorMatrix=pred.m2, method="cart",
                           minbucket=mb, cp=cpm, printFlag=FALSE)
  tmp.scenario1 <- mice::complete(tmp.mi.scenario1, 1)
  tmp.res1 <- subset(tmp.scenario1,
                     select=c('study','step',
                              colnames(tmp.scenario1)
                              [grep("\\.7$",colnames(tmp.scenario1))]))
}

#Scenario 2:
if(scenario2){
  pred.m2 <- pred.m1
  #living_status and outcome(s) not imputed and not used as predictors:
  pred.m2[,paste0(c('living_status.',paste0(outcome.v,'.')),n.waves)]
  <- 0
  pred.m2[paste0(c('living_status.',paste0(outcome.v,'.')),n.waves),]
  <- 0
  imp.data <- is.na(tmp)
  imp.data[,paste0(c('living_status.',paste0(outcome.v,'.')),n.waves)]
```

```
<- FALSE

tmp.mi.scenario2 <- mice(tmp, m=1, maxit=10, predictorMatrix=pred.m2,
                         where=imp.data, method="cart", minbucket=mb,
                         cp=cpm, printFlag=FALSE)
tmp.scenario2 <- mice::complete(tmp.mi.scenario2, 1)

sd.hrs.a <-
  with(subset(tmp.scenario2,step==1&study=='HRS'),
       sd(adl_score.1))
sd.elsa.a <-
  with(subset(tmp.scenario2,step==1&study=='ELSA'),
       sd(adl_score.2))
sd.h2000.a <-
  with(subset(tmp.scenario2,step==1&study=='Health2000-2011'),
       sd(adl_score.1))
sd.hrs.b <-
  with(subset(tmp.scenario2,step==1&study=='HRS'),
       sd(iadl_score.1))
sd.elsa.b <-
  with(subset(tmp.scenario2,step==1&study=='ELSA'),
       sd(iadl_score.2))
sd.h2000.b <-
  with(subset(tmp.scenario2,step==1&study=='Health2000-2011'),
       sd(iadl_score.1))

#define scenario 2 (by cohort):
sds.adl <- c(sd.hrs.a, sd.elsa.a, sd.h2000.a)
sds.iadl <- c(sd.hrs.b, sd.elsa.b, sd.h2000.b)
sts <- c('HRS','ELSA','Health2000-2011')
for(i in 1:3){
  tmp.subset <- tmp.scenario2$step==2 &
    tmp.scenario2[[paste0('vig_pa.',n.waves)]]=='No' &
    tmp.scenario2$study==sts[i]
  tmp.scenario2[[paste0('adl_score.',n.waves)]][tmp.subset] <-
    round(rnorm(length(tmp.scenario2[[paste0('adl_score.',n.waves)]]
                       [tmp.subset]),
                mean=tmp.scenario2[[paste0('adl_score.',n.waves)]]
                [tmp.subset]+(1.12*sds.adl[i]), sd=sds.adl[i]))
  tmp.scenario2[[paste0('iadl_score.',n.waves)]][tmp.subset] <-
    round(rnorm(length(tmp.scenario2[[paste0('iadl_score.',n.waves)]]
                       [tmp.subset]),
                mean=tmp.scenario2[[paste0('iadl_score.',n.waves)]]
                [tmp.subset]+(1.12*sds.iadl[i]), sd=sds.iadl[i]))
}
tmp.scenario2[[paste0('adl_score.',n.waves)]]
[tmp.scenario2$step==2 &
    tmp.scenario2[[paste0('adl_score.',n.waves)]]>6] <- 6
tmp.scenario2[[paste0('adl_score.',n.waves)]]
[tmp.scenario2$step==2 &
    tmp.scenario2[[paste0('adl_score.',n.waves)]]<0] <- 0
tmp.scenario2[[paste0('iadl_score.',n.waves)]]
[tmp.scenario2$step==2 &
    tmp.scenario2[[paste0('iadl_score.',n.waves)]]>6] <- 6
tmp.scenario2[[paste0('iadl_score.',n.waves)]]
[tmp.scenario2$step==2 &
    tmp.scenario2[[paste0('iadl_score.',n.waves)]]<0] <- 0

pred.m2 <- pred.m1
#pa not used as a predictor for outcome(s) and mortality:
pred.m2[,paste0('vig_pa.',n.waves)] <- 0
```

```
    tmp.mi.scenario2 <- mice(tmp.scenario2, m=1, maxit=10,
                             predictorMatrix=pred.m2, method="cart",
                             minbucket=mb, cp=cpm, printFlag=FALSE)
    tmp.scenario2 <- mice::complete(tmp.mi.scenario2, 1)
    tmp.res2 <- subset(tmp.scenario2,
                       select=c('study','step',
                                colnames(tmp.scenario2)
                                [grep("\\.7$",colnames(tmp.scenario2))]))
  }
  rm(tmp); gc()
}

if(i.pred>2){
  if(scenario0){
    tmp02 <- subset(tmp.scenario0, as.numeric(step)==(i.pred-1) &
                      living_status.7=='Alive', select=-c(step))
    tmp02 <- tmp02[,!grepl("\\.1$", colnames(tmp02))]
    colnames(tmp02) <- unlist(lapply(
      strsplit(colnames(tmp02),'[.]'), function(a)
        ifelse(length(a)<2,a,paste0(a[1],'.',as.numeric(a[2])-1))))
    tmp.scenario0 <- rbind.fill(tmp.scenario0, cbind(step=i.pred, tmp02))
    rm(tmp02); gc()
    ## Make some transformations e.g. add age some constant,
    ## copy sex the same value etc
    tmp.scenario0 <-
      rbind(subset(tmp.scenario0, as.numeric(step) < i.pred),
            transf.fn2(subset(tmp.scenario0, as.numeric(step) == i.pred),
                       n.waves, years.between.waves, education = TRUE))
    incl.steps <- c(i.pred-1,i.pred)
    tmp.scenario0 <- subset(tmp.scenario0,as.numeric(step)%in%incl.steps)

    tmp.mi <- mice(tmp.scenario0, m=1, maxit=10, predictorMatrix=pred.m1,
                   method="cart", minbucket=mb, cp=cpm, printFlag=FALSE)
    tmp.scenario0 <- mice::complete(tmp.mi, 1)
    tmp.res0 <-
      rbind(tmp.res0,subset(
        tmp.scenario0,as.numeric(step)==i.pred,
        select=c('study','step',colnames(tmp.scenario0)
                 [grep("\\.7$", colnames(tmp.scenario0))])))
  }
  if(scenario1){
    tmp12 <- subset(tmp.scenario1, as.numeric(step)==(i.pred-1) &
                      living_status.7=='Alive', select=-c(step))
    tmp12 <- tmp12[,!grepl("\\.1$", colnames(tmp12))]
    colnames(tmp12) <- unlist(lapply(
      strsplit(colnames(tmp12),'[.]'), function(a)
        ifelse(length(a)<2,a,paste0(a[1],'.',as.numeric(a[2])-1))))
    tmp.scenario1 <- rbind.fill(tmp.scenario1, cbind(step=i.pred, tmp12))
    rm(tmp12); gc()
    ## Make some transformations e.g. add age some constant,
    ## copy sex the same value etc
    tmp.scenario1 <-
      rbind(subset(tmp.scenario1, as.numeric(step) < i.pred),
            transf.fn2(subset(tmp.scenario1, as.numeric(step) == i.pred),
                       n.waves, years.between.waves, education = TRUE))
    incl.steps <- c(i.pred-1,i.pred)
    tmp.scenario1 <- subset(tmp.scenario1,as.numeric(step)%in%incl.steps)

    tmp.mi <- mice(tmp.scenario1, m=1, maxit=10, predictorMatrix=pred.m1,
                   method="cart", minbucket=mb, cp=cpm, printFlag=FALSE)
```

```
      tmp.scenario1 <- mice::complete(tmp.mi, 1)
      tmp.res1 <-
        rbind(tmp.res1,subset(
          tmp.scenario1,as.numeric(step)==i.pred,
          select=c('study','step',colnames(tmp.scenario1)
                   [grep("\\.7$", colnames(tmp.scenario1))])))

    }
    if(scenario2){
      tmp22 <- subset(tmp.scenario2, as.numeric(step)==(i.pred-1) &
                        living_status.7=='Alive', select=-c(step))
      tmp22 <- tmp22[,!grepl("\\.1$", colnames(tmp22))]
      colnames(tmp22) <- unlist(lapply(
        strsplit(colnames(tmp22),'[.]'), function(a)
          ifelse(length(a)<2,a,paste0(a[1],'.',as.numeric(a[2])-1))))
      tmp.scenario2 <- rbind.fill(tmp.scenario2, cbind(step=i.pred, tmp22))
      rm(tmp22); gc()
      ## Make some transformations e.g. add age some constant,
      ## copy sex the same value etc
      tmp.scenario2 <-
        rbind(subset(tmp.scenario2, as.numeric(step) < i.pred),
              transf.fn2(subset(tmp.scenario2, as.numeric(step) == i.pred),
                         n.waves, years.between.waves, education = TRUE))
      incl.steps <- c(i.pred-1,i.pred)
      tmp.scenario2 <- subset(tmp.scenario2,as.numeric(step)%in%incl.steps)

      tmp.mi <- mice(tmp.scenario2, m=1, maxit=10, predictorMatrix=pred.m1,
                     method="cart", minbucket=mb, cp=cpm, printFlag=FALSE)
      tmp.scenario2 <- mice::complete(tmp.mi, 1)
      tmp.res2 <- rbind(tmp.res2,subset(
        tmp.scenario2,as.numeric(step)==i.pred,
        select=c('study','step',colnames(tmp.scenario2)
                 [grep("\\.7$", colnames(tmp.scenario2))])))
    }
  } #i.pred>2

} #for i.pred

#only step transformed back to numeric:
if(pred.steps>1){
  if(scenario0) tmp.res0[,'step'] <-
      as.numeric(as.character(tmp.res0[,'step']))
  if(scenario1) tmp.res1[,'step'] <-
      as.numeric(as.character(tmp.res1[,'step']))
  if(scenario2) tmp.res2[,'step'] <-
      as.numeric(as.character(tmp.res2[,'step']))
}

if(scenario0){
  res0[[i.b]] <- tmp.res0; rm(tmp.scenario0)
}
if(scenario1){
  res1[[i.b]] <- tmp.res1; rm(tmp.scenario1)
}
if(scenario2){
  res2[[i.b]] <- tmp.res2; rm(tmp.scenario2)
}

imp0[[i.b]] <- tmp.imp
if(pred.steps==1) predm1[[i.b]] <- pred.m
if(pred.steps>1)  predm1[[i.b]] <- pred.m1
```

```
    rm(pred.m, pred.m1, tmp.all, tmp.imp, tmp.res0, tmp.res1, tmp.res2)
    gc()
  }

  rm(bs, tmp0); gc()

  if(!scenario0)
    return(list("imp0"=imp0,"predm1"=predm1)) #no scenarios, pred.steps=1
  if(scenario0 & !scenario1 & !scenario2)
    return(list("res0"=res0,"imp0"=imp0,"predm1"=predm1))
  if(scenario0 & scenario1 & !scenario2)
    return(list("res0"=res0,"res1"=res1,"imp0"=imp0,"predm1"=predm1))
  if(scenario0 & !scenario1 & scenario2)
    return(list("res0"=res0,"res2"=res2,"imp0"=imp0,"predm1"=predm1))
  if(scenario0 & scenario1 & scenario2)
    return(list("res0"=res0,"res1"=res1,"res2"=res2,"imp0"=imp0,
                "predm1"=predm1))
}
```

REFERENCES

1. Caballero FF, Soulis G, Engchuan W, Sánchez-Niubó A, Arndt H, Ayoso-Mateos JL, et al. Advanced analytical methodologies for measuring healthy ageing and its determinants, using factor analysis and machine learning techniques: the ATHLOS project. Sci Rep. 2017; 7:43955. https://doi.org/10.1038/srep43955.
2. Hänninen T, Pulliainen V, Sotaniemi M, Hokkanen L, Salo J, Hietanen M, et al. Muistisairauksien tiedonkäsittelymuutosten varhainen toteaminen: Uudistettu CERAD-tehtäväsarja (Early detection of cognitive changes in memory diseases: New cut-off scores for the Finnish version of the CERAD neuropsychological battery). Duodecim. 2010; 126(17):2013–2021.
3. Breiman L, Friedman JH, Olshen RA, Stone CJ. Classification and Regression Trees. Monterey (CA): Wadsworth & Brooks/Cole Advanced Books & Software; 1984.
4. van Buuren S, Groothuis-Oudshoorn K. mice: Multivariate Imputation by Chained Equations in R. J Stat Softw. 2011; 45(3):1–67. http://dx.doi.org/10.18637/jss.v045.i03.
5. R Core Team. R: A Language and Environment for Statistical Computing. Vienna, Austria: R Foundation for Statistical Computing; 2020.
6. van Buuren S, Groothuis-Oudshoorn K, Vink G, Schouten R, Robitzsch A, Rockenschaub P, et al. Package ‘mice’. CRAN; 2021. Available from: https://cran.r-project.org/web/packages/mice/mice.pdf. Accessed 15 September 2021.
7. Hadfield JD. MCMC Methods for Multi-Response Generalized Linear Mixed Models: The MCMCglmm R Package. J Stat Softw. 2010; 33:1–22. http://dx.doi.org/10.18637/jss.v033.i02.
8. Bernardo J, Smith AFM. Bayesian Theory. New York: Wiley; 1994.
9. Rubin DB. Multiple Imputation for Nonresponse in Surveys. New York: Wiley; 1987.
